# Refixation patterns reveal memory-encoding strategies in free viewing

**DOI:** 10.3758/s13414-019-01735-2

**Published:** 2019-05-01

**Authors:** Radha Nila Meghanathan, Andrey R. Nikolaev, Cees van Leeuwen

**Affiliations:** 1 Laboratory for Perceptual Dynamics, Brain & Cognition Research Unit, KU Leuven - University of Leuven, Tiensestraat 102, Box 3711, 3000 Leuven, Belgium; 2 Center for Cognitive Science, Technical University, Kaiserslautern, Germany

**Keywords:** Eye movements, Visual search, Embodied perception

## Abstract

We investigated visual working memory encoding across saccadic eye movements, focusing our analysis on refixation behavior. Over 10-s periods, participants performed a visual search for three, four, or five targets and remembered their orientations for a subsequent change-detection task. In 50% of the trials, one of the targets had its orientation changed. From the visual search period, we scored three types of refixations and applied measures for quantifying eye-fixation recurrence patterns. Repeated fixations on the same regions as well as repeated fixation patterns increased with memory load. Correct change detection was associated with more refixations on targets and less on distractors, with increased frequency of recurrence, and with longer intervals between refixations. The results are in accordance with the view that patterns of eye movement are an integral part of visual working memory representation.

**Electronic supplementary material:**

The online version of this article (10.3758/s13414-019-01735-2) contains supplementary material, which is available to authorized users.

## Introduction

Eye movements are generally executed to identify and encode new information (Henderson & Hollingworth, 1999; Irwin, 2004). Nevertheless, in real-world scenes, the eyes frequently perform *refixations*, that is, they return to previously fixated objects or locations (Beck, Peterson, & Vomela, 2006; Dickinson & Zelinsky, 2007; Zelinsky, Loschky, & Dickinson, 2011). Refixations may function to update a previous object description (Tatler, Gilchrist, & Land, 2005) or to rehearse an object in working memory (Zelinsky et al., 2011). Increases in refixation rates may be caused by insufficient processing of information at fixation (Droll & Hayhoe, 2007; Körner & Gilchrist, 2007; McCarley, Wang, Kramer, Irwin, & Peterson, 2003; Peterson, Beck, & Wong, 2008), for instance, due to premature shifts of attention prompted by high working memory load (Gilchrist & Harvey, 2000; Gilchrist, North, & Hood, 2001; Peterson, Kramer, Wang, Irwin, & McCarley, 2001).

Invariably, in these cases, refixations are made for acquisition of information that is deemed lost or incomplete. If this is the only reason why refixations are made, this would essentially be a random process. However, as we will argue, it is possible that some long-term, strategic processes underlie refixation behavior. We will consider the possibility that such processes are revealed in refixation behavior.

To explain how missing information triggers a refixation in visual memory tasks, Zelinsky et al. (2011) proposed a “monitor-refixate-rehearse” model. Representations of multiple fixated items are maintained in working memory. The item to be fixated next is determined by three factors – inhibition of return (Klein, 1988; Posner & Cohen, 1984), which draws gaze away from recently visited items; distance bias, which directs gaze to nearby items than to distant ones (Dickinson & Zelinsky, 2007; Findlay & Brown, 2006; Loschky & McConkie, 2002; Motter & Belky, 1998; Takeda & Yagi, 2000); and, last but not least, a visual working memory (VWM)-related component. This component directs gaze to items with fading activations. Zelinsky et al. (2011) proposed that VWM representations decrease in activation as the number of intervening items fixated increases.

Zelinsky's et al. (2011) model does not address the contents of VWM representations. VWM is generally considered to contain two types of information – identity and spatial location (reviewed in Baddeley & Logie, 1999; Beck et al., 2006; Smith & Jonides, 1997). Depending on the task, memory for either identity or location may play the predominant role in determining task performance, which, in turn, would affect refixation behavior. In visual search, object location rather than identity guides search (Beck et al., 2006). This would imply that memory for item location determines refixation behavior in visual search.

We may wonder whether, in addition to the spatial position of items, the temporal order of item visitation may also be part of the visual working memory representation. A serial position effect is seen for spatial memory (Smyth & Scholey, 1996; Zelinsky & Loschky, 2005), that is, items fixated first and last are recalled better. Current ideomotor theory (Hommel, Müsseler, Aschersleben, & Prinz, 2001) claims that perception shares a common code with motor behavior: motor actions are represented by their perceivable effects (Shin, Proctor, & Capaldi, 2010). Specifically for oculomotor behavior, this theory would imply that the eye-movement trajectory is memorized. Noton and Stark (1971) first described repetition of scan-paths – the sequence of eye movements entailing scanning of a visual stimulus – during encoding and recognition of line drawings. This finding gave rise to the *scan-path theory* (Noton & Stark, 1971), which states that the encoding order of features of a visual stimulus is repeated during successful recognition of the same stimulus. Subsequent studies have shown that fixation sequences performed during encoding are repeated, not only during retrieval (Foulsham & Kingstone, 2013; Johansson, Holsanova, Dewhurst, & Holmqvist, 2012; Johansson & Johansson, 2014; Laeng et al., 2014; Valuch, Becker, & Ansorge, 2013; Wynn et al., 2016), but also for rehearsal of items in VWM after the stimuli have disappeared (Brandt & Stark, 1997; Brockmole & Irwin, 2005; Johansson et al., 2012; Laeng & Teodorescu, 2002; Spivey & Geng, 2001) or during the retention interval (Tremblay, Saint-Aubin, & Jalbert, 2006). Disruption of spontaneous fixation sequences interferes with VWM (Awh & Jonides, 2001), in particular for object details (Bochynska & Laeng, 2015; Johansson & Johansson, 2014; Laeng et al., 2014). Taken together, the above findings suggest that, in addition to identity and location, VWM representations may include information about the order in which items are visited. In particular, during encoding of visual stimuli in free viewing, scan-path information is likely encoded in VWM.

If scan-paths are part of the memory representation, it is possible that this information is actively used to promote refixation behavior. Refixation behavior may reflect encoding strategies used to memorize multiple targets in visual search. We therefore investigated refixations as a tool for studying the role of the scan-path in memorization, using a combined visual search/change detection task. During ten second periods, participants performed a visual search for 3, 4 or 5 targets and remembered their orientations for a subsequent change detection task.

Scan-paths or parts thereof encoded in VW, moreover, may be rehearsed during memorization. This implies that, in addition to identifying refixations on item locations, it is imperative to identify refixation *patterns*; that is, the occurrence of fixation sequences and their repetition later in time. To this end, we investigated how memory encoding depends on both refixations on specific items and refixation patterns under different memory load conditions. We assessed refixation behavior in memory encoding using two approaches: (1) identification of refixations separately on targets and distractors and (2) identification of refixation patterns.

For the former, we used a traditional method of counting refixations to objects. Since eye-movement behavior could be qualitatively different for targets and distractors (Körner et al., 2014; Meghanathan, van Leeuwen, & Nikolaev, 2015; Peterson et al., 2001), it is likely that refixation behavior will be different for the two. To take into account differences in target-distractor (re)fixation behavior, we counted refixations on targets and distractors separately. Moreover, we classified refixations into three types based on the numbers of intervening items. We analyzed the effect of memory load on these three types of refixations, for both targets and distractors. In line with previous studies, we expected to find increased target refixations as memory load increased (Körner & Gilchrist, 2007; McCarley et al., 2006; Peterson et al., 2008). In the same vein, we also expected to find increased distractor refixations with increase in memory load. Assuming refixations are performed for rehearsal of items, we expected that accurate trials would show higher number of refixations on targets than inaccurate trials.

In addition to the use of refixations motivated by scan-path information, we still must consider that target refixations may be made for maintenance of target identity in working memory (Zelinsky et al., 2011). The same is not likely for distractors, since task performance is not incumbent on distractor identity. Distractor refixations may be made in the event of forgetting of previously visited distractor locations. Given these contrasting causes of target and distractor refixations, their occurrence during search and encoding will change within trials over time in different ways, as more targets are found. Körner et al. (2014) showed that there was a difference in distractor processing before and after finding the first target in a visual search for two targets. Assessing how refixation behavior changes over the course of a trial would reveal how participants adapt encoding strategies for targets and distractors during the execution of the task. Therefore, we also counted refixations in time bins over the course of task performance and compared them across the three refixation types.

As counting the number of refixations cannot adequately estimate refixation patterns, as our second approach, we adopted recurrence quantification techniques (Anderson, Bischof, Laidlaw, Risko, & Kingstone, 2013; Webber & Zbilut, 2005) to identify patterns in fixation sequences. These techniques involve the use of recurrence plots, which describe trajectories of a dynamic system in state space. Every time a dynamic system revisits a state in state space, it is said to recur. Anderson et al. (2013) used recurrence quantification in eye movement behavior to study refixations. We adopted their four measures, namely, recurrence, laminarity, determinism, and center of recurrence mass (CORM). Briefly, recurrence is a measure of refixations, laminarity is a measure of refixation clusters, determinism measures repeating fixation sequences, and CORM measures temporal proximity between refixations (see *Material and methods* for details). In this analysis, we did not differentiate between targets and distractors. This *item-agnostic* analysis was performed, specifically, for identifying refixation patterns relevant to scan-path rehearsal. Our goals were to detect scan-path rehearsal, and to investigate the effect of memory load. We expected to find an increase in recurrence and laminarity indicating refixations to the same locations with an increase in memory load. We also expected an increase in determinism with increase in memory load, indicating an increase in scan-path rehearsal with memory load. We also evaluated the effectivity of these eye-movement strategies by comparing successful and unsuccessful trials. Because of the novelty of application of recurrence quantification measures for the refixation analysis, this part of our study was in part exploratory.

## Material and methods

We analyzed refixations in data from an EEG eye-tracking co-registration experiment, for which some results have been published earlier (Meghanathan et al., 2015; Nikolaev, Meghanathan, & van Leeuwen, 2016; Seidkhani et al., 2017; details of co-registration with EEG may be found in the preceding papers). This section emphasizes the methodological details relevant to the refixation analyses.

### Participants

Twenty-three participants (seven male) took part in the experiment. The departmental Ethics Committee of the KU Leuven had approved the study. All participants gave their written informed consent. Their mean age was 20.86 years (range 18–29 years). Of these, 15 reported normal vision, and six had their vision corrected to normal with eye-glasses and two with contact lenses. Data from two participants with noisy eye-movement recordings were excluded from analysis. Data from one more participant, who had no inaccurate trials in the three-target condition, were also excluded. Data from the remaining 20 participants (six male) are presented here.

### Stimuli

We presented a sequence of two displays per trial, each 39.9° × 30.5° of visual angle: a *search* display and a *change detection* display. Both displays contained 40 items, presented in black (0.48 cd/m^2^) against a gray background (32.84 cd/m^2^), placed randomly in each trial within a rectangular space of size 32.9° × 23.12°. Of these items, three, four, or five were target Ts and the remaining ones distractor Ls. Targets and distractors kept the same locations between the two displays. Targets (0.41° × 0.41°) and distractors (0.31° × 0.41°) occurred in various orientations, randomly chosen with equal probability, rotated by 20°, 80°, 140°, 200°, 260°, or 320° clockwise from the upright orientation. The targets in each display had different orientations. Items kept the same orientations between the two displays in a trial, except for one target in half of the trials, which was randomly assigned a different orientation. Each item was encircled (0.83°) to deter peripheral detection (Körner & Gilchrist, 2007; Peterson et al., 2001). No two items were closer than 3.12°, while targets were always separated by at least 6.24°.

### Apparatus and eye-movement recording

Stimuli were presented on a monitor (40cm × 30cm) with a refresh rate of 75 Hz and a screen resolution of 1,600 × 1,200 pixels. Participants were seated 55 cm from the monitor with their head stabilized on a chin rest. Eye movements were recorded using an SR Research Ltd. EyeLink 1000 video-based eye tracker.

The eye position was sampled at 250 Hz. Before the start of the experiment, a 9-point calibration was performed for the left eye at the center, four corners, and four mid-points along the edges. For participants with consistently poor calibration in the left eye, the right eye was tracked. For 14 of the 20 participants whose data are presented here, the left eye was tracked. Before each trial, drift correction was performed when error was less than 2°. Calibration was repeated if the error exceeded 2° and before each block of trials.

### Procedure

A *search* display was presented for 10 s. Participants were asked to search for three, four, or five target Ts among distractor Ls and memorize the orientation of the targets. After an inter-stimulus interval that randomly varied in duration between 1 s and 1.5 s, the *change detection* display was presented. Participants were asked to respond by pressing one of two alternative keys to indicate whether or not they detected a change. The change detection display was presented until key press or for a maximum duration of 10 s. Afterwards, a feedback screen was displayed, indicating all targets in green for a correct response or in red for an incorrect response, with the changed target surrounded by a large yellow circle. The experiment lasted around 100 min, during which participants performed 270 trials over ten blocks of 27 trials each, with short breaks between blocks. There were 90 trials in each target condition (three, four, or five), randomized across the blocks.

### Eye-movement data analysis

We obtained fixation durations and locations as output from the EyeLink software, based on an eye velocity threshold of 22°/s and acceleration of 3,800°/s^2^ for saccade detection. We only analyzed eye movements from the search display.

#### Item-based refixation analysis

**Fig. 1:**
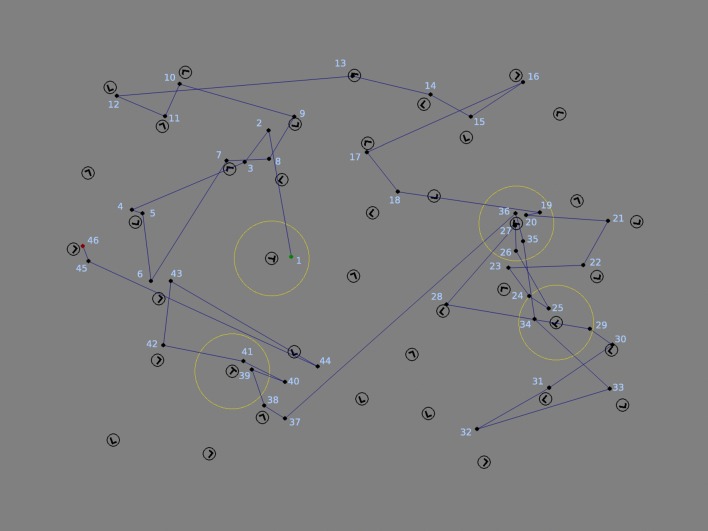
An example display with four targets and a fixation sequence in one 10-s trial. Fixations are shown as dots and are connected by lines indicating saccades. The first fixation of the sequence is in green and the last is in red. The yellow circles around target Ts indicate the 2° fixation criterion for assignment of fixations to targets. According to the criteria of Fig. 2, Fixation 20 is a continued refixation, Fixations 26, 29, 34, and 35 are revisits and Fixations 27 and 36 are continued revisits. Fixation 41 occurs after a fixation on empty space (Fixation 40), and is not counted as a revisit

We scored refixations on targets and distractors by counting the number of refixations on each item. We only considered fixations shorter than or equal to 1,000 ms, which comprised 99.9% of the total number of fixations. For each trial, fixation sequences were assessed such that each fixation was assigned to a target, distractor, or neither, depending on a distance criterion. On average, 8% of fixations were assigned to neither target nor distractor. The distance criterion required a fixation on a target or distractor to be within 2° of visual angle from the center of an item on the display. This 2° criterion was chosen to ensure that the distance between a fixation and refixation stayed within the diameter of the fovea, in agreement with previous studies (McCarley et al., 2003; Peterson et al., 2001). As an example, a fixation sequence and the 2° criterion for targets overlaid on the display of a trial with four targets are shown in Fig. 1.

**Fig. 2:**
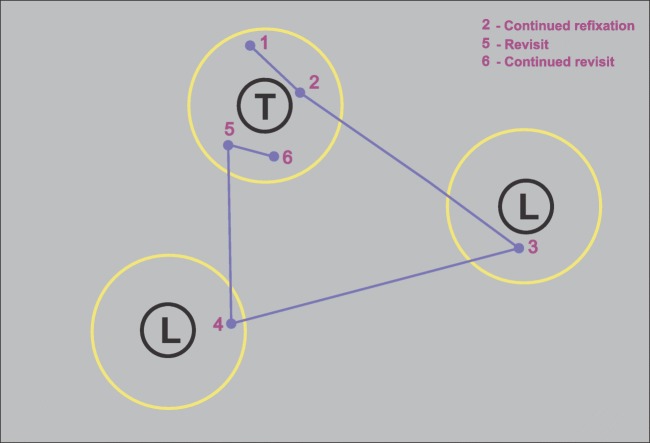
Illustration of refixation types in a sample fixation sequence. The fixation sequence is illustrated with purple dots connected by lines with the fixation numbers written next to the fixations. A target T and two distractor Ls are shown. The 2° criterion is indicated by yellow circles around each item. Fixations 1, 2, 5, and 6 all occur on the target T. Fixation 1 is a first fixation on the target. Fixation 2 occurs after Fixation 1 without leaving the yellow region, and is therefore a continued refixation. Fixation 5 occurs after leaving the yellow region, following intervening Fixations 3 and 4, and is hence a revisit. Fixation 6 is a continued revisit

When an item in a given display was fixated more than once, all subsequent fixations on that item were counted as refixations. We considered three different types of refixations, as illustrated in Fig. 2. *Continued refixations* are ones that occur within the 2° boundary of the item region before leaving it for the first time, as illustrated in Fig. 2 by Fixation 2. Refixations after leaving the item region are classified as *revisits* if they arrive from outside the region (Fixation 5 in Fig. 2). *Continued revisits* are refixations from within the item region after it had been left at least once, as illustrated by Fixation 6 in Fig. 2. Refixations from outside of item regions, that is, on empty spaces in the display, were not included in the current classification. Figure 1 illustrates the above classification of refixations in the display of a trial. Note that, by definition, revisits are preceded by larger saccades than continued refixations and continued revisits (ref. Fig. 5 and Table 1).

**Table 1.** Mean preceding saccade sizes (in degrees of visual angle) and fixation durations (in ms) with standard deviations in parentheses of continued refixations, revisits, and continued revisits on targets and distractors

	Saccade Size			Fixation Duration		
	Continued refixations	Revisits	Continued revisits	Continued refixations	Revisits	Continued revisits
Targets	2.4 (0.9)	6.4 (0.6)	1.4 (0.3)	234.1 (61.9)	222.3 (25.5)	203.6 (34)
Distractors	1.6 (0.2)	5.6 (0.5)	1.5 (0.2)	150.1 (17.5)	186.4 (21.2)	151.8 (18.3)

Since target and distractor refixations may involve different underlying cognitive processes (Körner et al., 2014), refixation counts were analyzed separately for targets and distractors. Trials with accurate and inaccurate responses, moreover, may vary in the pattern of refixations. Therefore, we distinguished between the two in this analysis.

To assess the effect of memory load, each type of refixation count was separately averaged for accurate and inaccurate trials for each target condition for each participant. This yielded six different refixation counts for each target condition of three, four, and five targets. However, these fixation counts were not directly comparable because of the varying target-distractor ratios for displays in different trials. To correct for this difference, we divided the counts for targets by the number of targets in the display and the counts for distractors by the number of distractors.

Participants knew that three, four, or five targets may occur in a trial but did not know exactly how many targets to expect in each trial. This may have led to different search rates in each target condition. To rule out the possibility that differences in refixation counts between conditions were caused by the different rates of target discovery in the course of a search trial, we computed the time course of finding targets during a 10-s search interval separately for each target condition. We calculated the number of targets fixated within 1-s windows during the 10-s search interval. We called the obtained number “the cumulative target fixation score.” We averaged the cumulative target fixation scores across trials for each participant and averaged across participants (Fig. S1). The score shows that the rate at which targets were fixated was different for each target condition. Mid-way through the search interval, at 5 s, more than two targets were fixated in the four- and five-target conditions, whereas less than two targets had been fixated in the three-target condition. At the end of the 10-s interval, all the targets presented were barely found ruling out the possibility that in the conditions with more number of targets, targets were found earlier allowing for more time at the end of a trial for rehearsal or refixations.

In order to identify changes in memory-encoding strategies through the course of the search task, we compared refixation counts for each refixation type in 1-s windows of the 10-s search interval. Since refixation type and time progression during the search interval are dependent, the counts cannot be compared directly. By definition, revisits occur after continued refixations. In order to compare the different refixation types, we used the indirect approach of comparing each refixation type with refixation counts from scan-paths generated from a random model. To construct this random model, we first used a Gaussian kernel (σ = 10) to jitter the pool of target and distracter positions in each display for accurate trials. We only used accurate trials, since the number of inaccurate trials was insufficient to generate a reasonable number of random trials, which allows for averaging. Next, we selected as many values from the jittered pool at random as there were fixations in the search interval of the corresponding trial. These values comprised a pseudo-sequence of fixations for a trial. From this pseudo-sequence, we counted refixations of each type in the same fashion as for actual trials. We compared the refixation counts for each refixation type with those obtained for the random model separately for targets and distractors.

#### Recurrence analysis

**Fig. 3:**
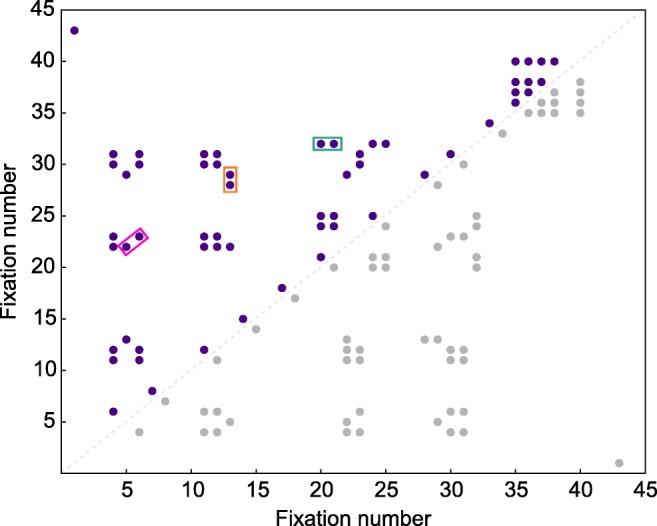
Recurrence plot of fixations in the 10-s visual search interval of a trial. Fixations are plotted against themselves in their order of occurrence in the search interval, such that a dot on this plot indicates a recurring fixation. The plot is symmetric along the central diagonal because fixations are plotted against themselves. Hence, useful information can be derived from either half of the plot. The redundant half is shown in gray here. Recurrence is computed as the percentage of recurring fixations in an interval. Determinism is calculated as the percentage of diagonal lines (pink) formed by pairs of two or more dots. Laminarity is computed as the proportion of vertical (orange) and horizontal (green) lines, each line being formed by at least two or more dots. Note that, to qualify as a repetition of fixation patterns, a line should be comprised of at least two dots

To identify refixation patterns, we computed recurrence quantification measures from recurrence plots (Anderson et al., 2013; Webber & Zbilut, 2005) based on the percentage of recurrences in a fixation sequence as shown in Fig. 3. In contrast to the item-based refixation analysis, these measures did not consider the proximity of a fixation to targets and distractors on the display, in order to avoid biases introduced in the item-based analysis. For instance, if a fixation falls outside the 2° criterion, it is not considered a fixation on the item. This may have led to loss of those fixations that occurred just outside the criterion. This problem is altogether avoided in the recurrence analysis by basing it only on the spatial location of items. Additionally, we were able to test our results against different radius criteria (from 0.5° to 3°) for recurrence by keeping it blind to target-distractor proximity. In the item-based analysis, such a test would have required further criteria for assigning a fixation to an item. Through this item-agnosticism, we were able to identify refixation patterns without superimposing interpretations based on area of interest on the refixations. This makes the recurrence analysis complementary to the item-based refixation analysis.

We used four measures, namely *recurrence*, *determinism*, *laminarity*, and *Center of Recurrence Mass (CORM)* as described by Anderson et al. (2013). *Recurrence* is computed as the percentage of fixations on the same location in the display of a trial. In a trial, a fixation is considered recurrent if a later fixation occurs within a distance criterion of 2° of the fixation. The *recurrence* of a trial is used to compute the other three measures. These measures were computed from patterns of recurrent points in the recurrence plot (Fig. 3). *Determinism* is a measure of repeated fixation trajectories, which is computed as the percentage of recurrent points forming diagonal lines on a recurrence plot. A diagonal line is comprised of at least two recurrent points and is formed when a fixation sequence is repeated. *Laminarity* identifies clusters of fixations repeated in time and is computed as the percentage of recurrent points forming horizontal and vertical lines on a recurrence plot. A horizontal or vertical line is comprised of at least two recurrent points. A horizontal line is formed when a fixation is recurrent with a set of earlier successive fixations, indicating that a region is first scanned in detail and then revisited briefly. A vertical line is formed when successive fixations are recurrent with a previous fixation indicating that a region that was briefly visited earlier is scanned in detail later. While the above described recurrence measures describe the patterns of refixations on regions in the display, the final measure, *CORM,* is a measure of the temporal proximity of recurrent points. This measure is computed by finding the distance of the center of mass of all recurring points from the central diagonal of the recurrence plot. A trial with higher *CORM* indicates that refixations occurred farther away in time from first fixations than in a trial with lower *CORM*. Note that regions in the recurrence analysis are not associated to items on the display (e.g., they do not distinguish target and distractor fixations) and are only defined by the spatial proximity of fixations.

**Fig. 4:**
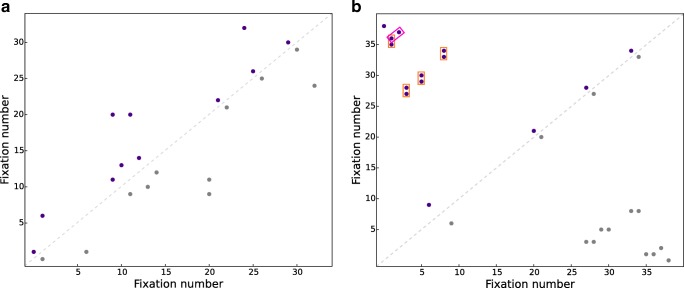
Recurrence plots for one participant in **a**: A trial with a recurrence value of 1.65, laminarity 0, determinism 0, and CORM of 12.2 and in **b**: A trial with a recurrence value of 1.6, laminarity 4.5, determinism 9.1, and CORM of 22. In line with Fig. 3, the purple dots on this plot indicate recurrent fixations. Redundant dots in the lower diagonal of this symmetric plot are marked in grey. The vertical (orange) lines are used to compute laminarity. The diagonal lines (pink) are used to compute determinism

Fixation sequences from the search interval of each trial were used to compute all four recurrence measures. Recurrence plots of a participant for two trials with different recurrence measures are shown in Fig. 4a and b, respectively.

These recurrence measures were separately averaged for accurate and inaccurate trials for each target condition. To make the recurrence measures comparable between participants, all four measures were normalized for each participant by computing z-scores.

In all statistical analyses, unless indicated otherwise, we used repeated-measures ANOVA. Huynh-Feldt corrections were applied whenever sphericity violations were encountered and partial η^2^ values are reported for effect sizes. *Post hoc* analyses were carried out using Fisher’s LSD test.

## Results

The average accuracy in the task was 78.3% for all trials (see Table 1 in Meghanathan et al., 2015). There was an effect of target condition on accuracy (F(2,38) = 30.3, p < 0.001, η^2^ = 0.96). Accuracy was higher in the three-target condition than in the four- and five-target conditions (*p* < 0.001).

The average saccade size was 4.82° and fixation duration was 199.98 ms. Both saccade size (*F*(2, 38) = 4.5, *p* = 0.03, η^2^ = 0.19) and fixation duration (*F*(2, 38) = 84.6, *p* < 0.001, η^2^ = 0.82) increased with the number of presented targets. In the three-, four-, and five-target conditions, saccade sizes were 4.7°, 4.8°, and 4.8° and fixation durations were 192.8 ms, 197.7 ms, and 203.7 ms, respectively.

### Item-based refixation analysis

First, we checked whether the number of targets presented affected the total number of fixations in a trial using a one-way ANOVA with three levels (three, four, and five targets). The number of fixations decreased (*F*(2, 38) = 89.6, *p* < 0.001, η^2^ = 0.86) with the number of targets (all post hoc *p* < 0.001). The mean numbers of fixations in trials of the three-, four-, and five-target conditions were 42.8, 41.8, and 40.6, respectively. We also assessed the effect of number of presented targets separately on the number of target and distractor fixations in a trial. For corrected counts, there was no effect of number of presented targets on the number of target fixations. However, the number of distractor fixations decreased (*F*(2, 38) = 403.9, *p* < 0.001 η^2^ = 0.95) with number of targets presented (all *post hoc p* < 0.001).^1^

**Fig. 5:**
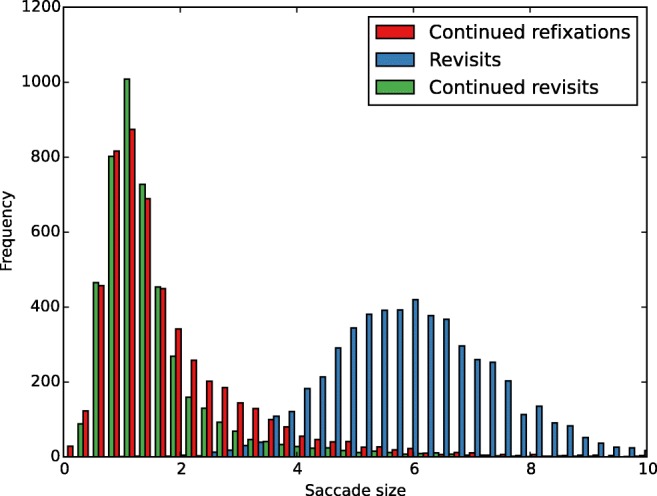
Histograms of saccade sizes (in degrees of visual angle) of continued refixations, revisits, and continued revisits for 20 participants

Next, we compared saccade sizes for the three refixation types (Table 1, Fig. 5). A repeated-measures ANOVA with factor refixation type (continued refixation, revisit, continued revisit) showed that refixation type had a significant effect on saccade size for both targets (*F*(2, 38) = 815.1, *p* < 0.001, η^2^ = 0.98) and distractors (*F*(2, 38) = 1158.6, *p* < 0.001, η^2^ = 0.98). The differences between all the fixation types were significant (all *p* < 0.001) for targets. For distractors, there was a significant difference between all fixation types (all *p* < 0.001) except between continued refixations and continued revisits.

We also compared fixation durations for the three refixation types (Table 1) in a repeated-measures ANOVA with factor refixation type (continued refixation, revisit, continued revisit). There was a significant effect of refixation type for both targets (*F*(2, 38) = 8, *p* = 0.001, η^2^ = 0.3) and distractors (*F*(2, 38) = 283.7, *p* < 0.001, η^2^ = 0.94). *Post hoc* tests showed no significant difference between the refixation types. For distractors, there was a significant difference between all refixation types (*p* < 0.001) except between continued refixations and continued revisits.

To assess the effect of memory load on refixation count, we performed a 2 × 3 × 3 repeated-measures ANOVA with factors accuracy (accurate, inaccurate), refixation type (continued refixations, revisits and continued revisits) and target condition (three, four, and five targets presented) on the *corrected* scores of refixation count, separately for targets and distractors.

**Fig. 6:**
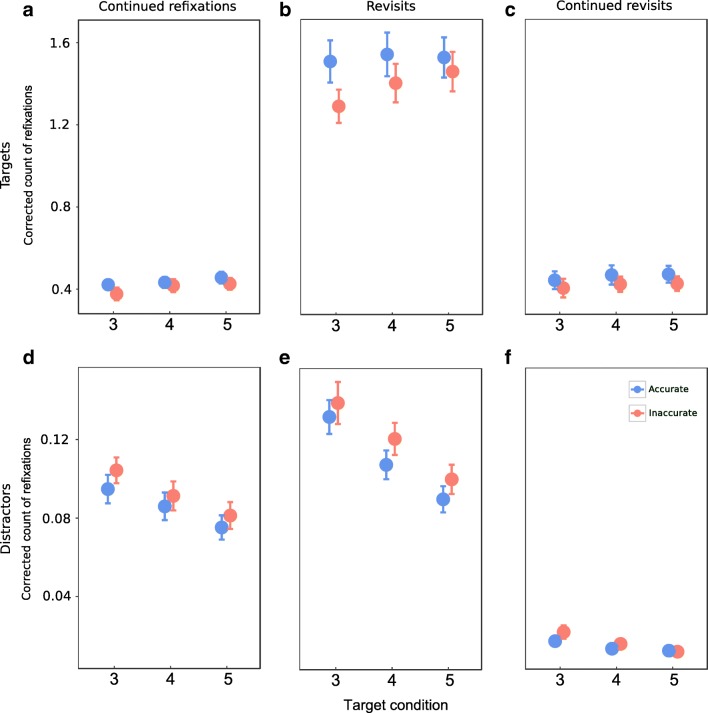
Three types of refixations in three-, four-, and five-target conditions. Corrected count of **a**: continued refixations on targets, **b**: revisits on targets, **c**: continued revisits on targets, **d**: continued refixations on distractors, **e**: revisits on distractors, and **f**: continued revisits on distractors. The three-, four-, and five-target conditions are along the x-axis. Data points are the means and error bars are the standard errors across 20 participants

Refixations showed an effect of accuracy on both targets (*F*(1, 19) = 12.8, p = 0.002, η^2^ = 0.4) and distractors (*F*(1, 19) = 34.5, p < 0.001, η^2^ = 0.64) (Fig. 6 with corrected scores, Fig. S2 with uncorrected scores). Refixation count was higher in accurate than inaccurate condition for targets, while the opposite was seen in distractors.

The main effects of refixation type on refixation count were significant for both targets (*F*(2, 38) = 115.1, p < 0.001, η^2^ = 0.86) and distractors (*F*(2, 38) = 131.7, p < 0.001, η^2^ = 0.87). There were more revisits than continued refixations and continued revisits for both targets (all *post hoc p* < 0.001) and distractors (all *post hoc p* < 0.001). There was no difference between the number of continued refixations and continued revisits for targets (Fig. 6a and c). On the other hand, there were more continued refixations than continued revisits for distractors (*post hoc p* < 0.001), as seen in Fig. 6d and f.

There were main effects of target condition on refixation count for both targets (*F*(2, 38) = 7.6, p = 0.003, η^2^ = 0.29) and distractors (*F*(2, 38) = 90.3, p < 0.001, η^2^ = 0.83). For targets, *post hoc* tests revealed lower refixation counts in the three-target condition than in the four-target (*p* = 0.008) and five-target (*p* < 0.001) conditions. For distractors, refixation count decreased with the number of presented targets (all *post hoc p* < 0.001).

There were interactions between refixation type and accuracy for both targets (*F*(2, 38) = 12.6, p < 0.001, η^2^ = 0.4) and distractors (*F*(2, 38) = 5.9, p = 0.01, η^2^ = 0.24). Specifically, for targets there were more revisits (*p* < 0.001) in accurate than inaccurate trials, while the opposite was observed for distractors (*p* < 0.001). Moreover, for targets alone, there were more continued revisits in accurate than inaccurate trials (*p* = 0.02). For distractors, there were more continued refixations in inaccurate trials than in accurate trials (*p* < 0.001). For both accurate and inaccurate trials, the nature of the difference between the refixation types for targets and distractors was the same as seen before: revisits were more than both continued refixations and continued revisits for targets (all *p* < 0.001) and distractors (all *p* < 0.001). Additionally, for distractors, continued refixations were more in number than continued revisits (all *p* < 0.001).

There was an interaction between refixation type and target condition for distractors alone (*F*(4, 76) = 24.1, *p* < 0.001, η^2^ = 0.56). Continued refixations and revisits decreased with the number of targets presented (all *p* < 0.001). There were more continued revisits in the three-target condition than in the four-target (*p* = 0.04) and five-target conditions (*p* = 0.002).

A significant interaction between refixation type, accuracy, and target condition was also found for targets (*F*(4, 76) = 3.2, *p* = 0.02, η^2^ = 0.14), while this effect was not found for distractors.

Thus, the opposite trend for target and distractor refixations with increasing number of targets on the display indicates that oculomotor strategies are adapted with increase in memory load. Moreover, the higher refixation count in accurate than inaccurate trials for targets and the opposite effect for distractors indicate that higher target refixations and lower distractor refixations are associated with enhanced performance. Specifically, we find that revisits are higher in the accurate trials than inaccurate trials for targets with the opposite being true for distractors. Moreover, there are also more continued revisits on targets in the accurate trials than inaccurate trials, whereas for distractors there are more continued refixations in inaccurate trials than accurate trials. The refixation types also differ between targets and distractors, with distractors alone showing more continued refixations than continued revisits.

Next, we assessed refixation behavior over the course of a search interval in ten 1-s windows. Since the counts of refixation types are dependent on each other, we compared refixation counts with those generated in a random model. We compared refixations in actual trials with those in the random model by computing a repeated measures 2 × 3 × 10 ANOVA with factors of type of data (actual, model), refixation type (continued refixations, revisits and continued revisits), and time window (ten levels), separately for targets and distractors. Refixation counts were pooled across three-, four-, and five-target conditions so that the number of trials in each time window would not be too small. Since we generated model data only from accurate trials, we only compared accurate trials from actual data with the model data.

**Fig. 7:**
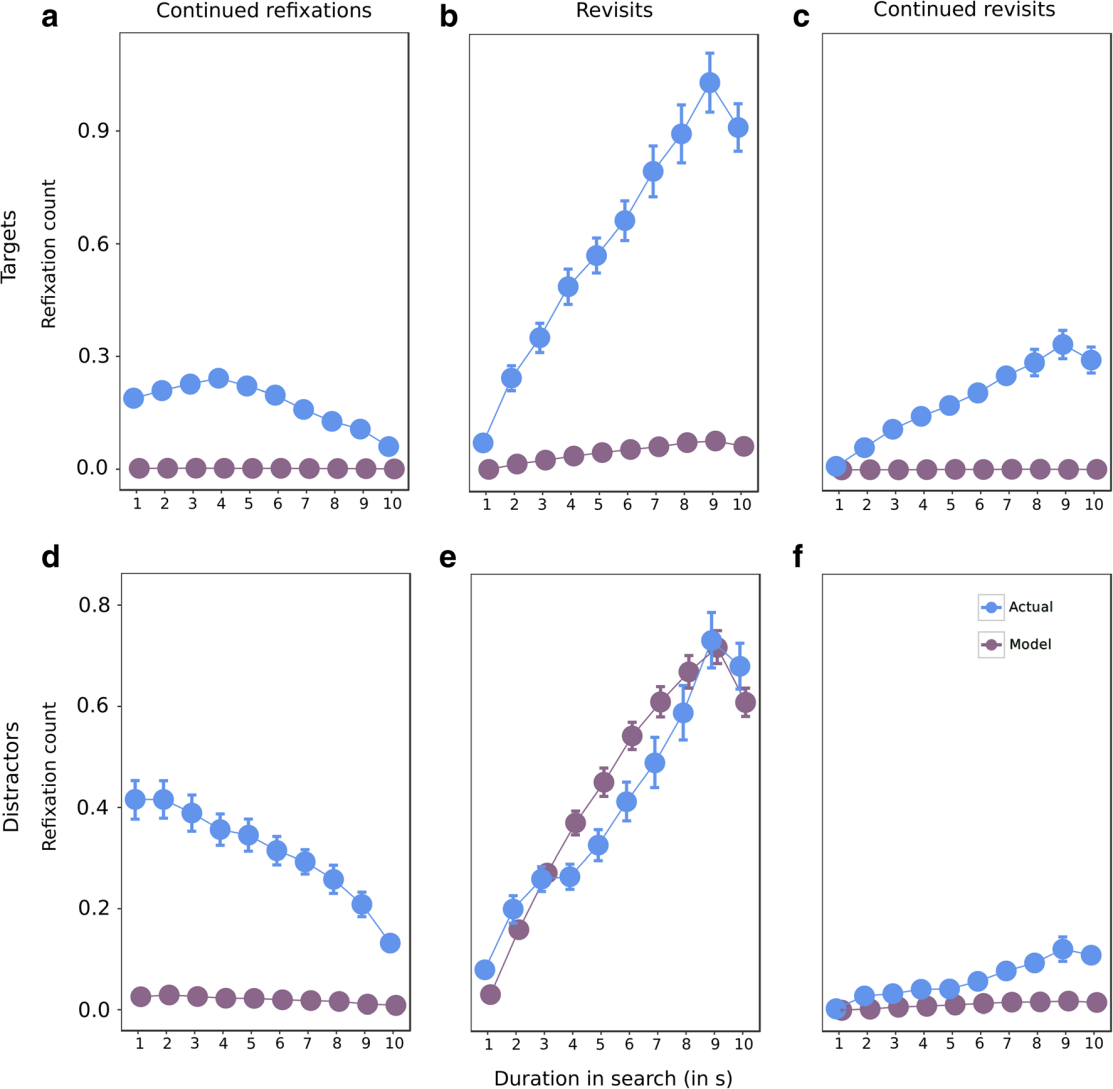
Time course of refixations within the 10-s interval of one search interval. Z-scores of count of refixations in 1-s time-windows for **a**: continued refixations on targets, **b**: revisits on targets, **c**: continued revisits on targets, **d**: continued refixations on distractors, **e**: revisits on distractors and **f**: continued revisits on distractors. The ten time windows are along the x-axis. Data points are the means and error bars are the standard errors across 20 participants

The ANOVA revealed a significant effect of time on refixation count of targets (*F*(9, 171) = 69.3, *p* < 0.001, η^2^ = 0.78) and distractors (*F*(9, 171) =123.9, *p* < 0.001, η^2^ = 0.87) (Fig. 7). There was also a significant effect of the type of data on refixations on targets (*F*(2, 38) = 326.8, *p* < 0.001, η^2^ = 0.94) and distractors (*F*(2, 38) = 84.9, *p* < 0.001, η^2^ = 0.82). There were more refixations in actual data compared to the model data. Refixation type also had a significant effect on both target refixations (*F*(2, 38) = 141.1, *p* < 0.001, η^2^ = 0.88) and distractor refixations (*F*(2, 38) = 295.2, *p* < 0.001, η^2^ = 0.94). There were more revisits than continued refixations and continued revisits for both targets and distractors. There were more continued refixations than continued revisits for distractors alone.

There was an interaction between time and data type for both target (*F*(9, 171) = 53.5, *p* < 0.001, η^2^ = 0.74) and distractor refixations (*F*(9, 171) = 10.9, *p* < 0.001, η^2^ = 0.36). There was also an interaction between time and refixation type for refixations on targets (*F*(18, 342) = 91.6, *p* < 0.001, η^2^ = 0.83) and distractors (*F*(18, 342) = 163.4, *p* < 0.001, η^2^ = 0.89). The interaction between data type and refixation type was also significant for target (*F*(2, 38) = 96.4, *p* < 0.001, η^2^ = 0.83) and distractor refixations (*F*(2, 38) = 115.9, *p* < 0.001, η^2^ = 0.86). There were more revisits than continued refixations and continued revisits for both actual and model data for both targets (all *p* < 0.01) and distractors (all *p* < 0.001). The number of refixations was higher in actual data than in model data for all three refixation types (*p* < 0.001) for target refixations. As seen in Fig. 7b, this difference was particularly pronounced for revisits. However, for distractor refixations, the number of revisits was higher in model data than in actual data (*p* = 0.01), whereas the opposite was seen for continued refixations and continued revisits (both *p* < 0.001). Fig. 7e shows that the higher revisits in model data than actual data are observed from about 4 s to 8 s. This difference is, however, marginal compared to the difference in revisits between model and actual data for target refixations (Fig. 7b). There was also a significant interaction between time, data type, and refixation type for targets (*F*(18, 342) = 67.8, *p* < 0.001, η^2^ = 0.78) and distractors (*F*(18, 342) = 21.8, *p* < 0.001, η^2^ = 0.53).

In a separate analysis, we averaged the accurate and inaccurate trials and compared the averaged values to model data. The results were similar to the analysis involving accurate trials alone.

In sum, the target-distractor differences between the refixation types were preserved. There were more target refixations in actual data than in model data for all three refixation types. However, the number of distractor revisits were as good as random. Continued refixations and continued revisits were higher in actual data than model data.

### Recurrence analysis

For each participant, we generated recurrence plots for every trial from which we computed recurrence, determinism, laminarity, and CORM measures. We validated all the computed recurrence measures against chance levels, as suggested by Anderson et al. (2013). The validation against chance levels is necessary because all recurrence measures for a trial are derived from the number of recurrent fixations in the trial, which is based on an arbitrary distance criterion (2°). Moreover, determinism and laminarity also depend on the choice of the number of repetitions of a gaze pattern (two in our case). To validate the recurrence measures, we generated a fixation model by applying a Gaussian filter (σ = 2.5) to all the fixations across all trials for each participant. From this distribution, we generated pseudo-fixation sequences for each trial 1,000 times to derive bootstrapped distributions for each of the four recurrence measures. Then we compared the bootstrapped sample of each recurrence measure for each participant with the empirically derived measures for each target condition using t-tests. We found that our measures were significantly above chance level measures obtained from bootstrapping (all p < 0.001).

**Fig. 8:**
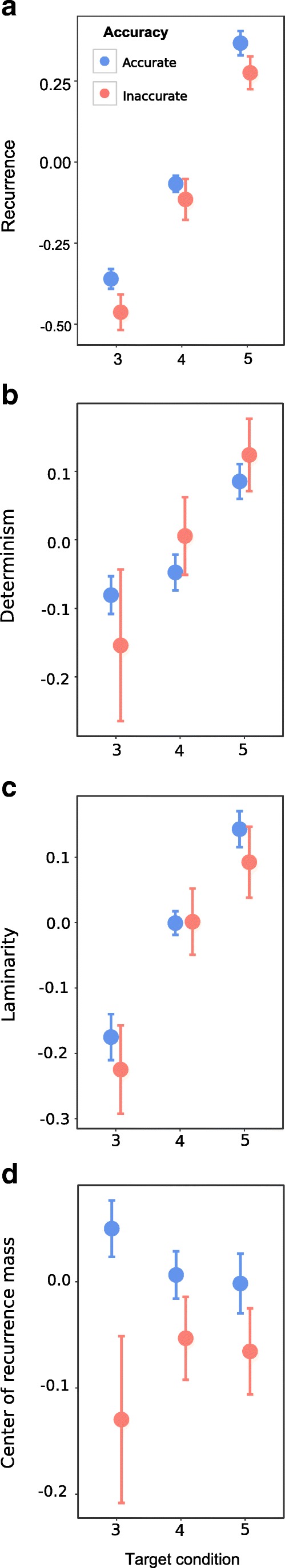
**a**: Recurrence, **b**: Determinism, **c**: Laminarity, and **d**: CORM, for three-, four-, and five- target conditions in accurate and inaccurate trials. The measures are normalized for each participant. Data points are the means and error bars are the standard errors across 20 participants

We performed 2 × 3 repeated-measures ANOVAs with factors accuracy (accurate, inaccurate) and target condition (three, four, and five targets presented) on each recurrence measure separately. Recurrence showed an effect of target condition (*F*(2, 38) = 125, *p* < 0.001, η^2^ = 0.87). *Post hoc* tests showed that recurrence increased with the number of targets (all *p* < 0.001) (Fig. 8a). Determinism also showed a target effect (*F*(2, 38) = 5.7, *p* = 0.009, η^2^ = 0.23). The five-target condition showed more determinism than the three-target condition (*p* < 0.001) (Fig. 8b) indicating an increase in repetition of fixation sequences with higher number of targets. There was no difference between the three- and four-target conditions and four- and five-target conditions. There was an effect of target condition on laminarity (*F*(2, 38) = 23.1, *p* < 0.001, η^2^ = 0.55). Laminarity increased with the number of targets (all *p* < 0.05) (Fig. 8c) indicating higher number of refixation clusters with higher number of targets. Only the CORM measure showed an effect of accuracy (*F*(1, 19) = 12.5, *p* = 0.002, η^2^ = 0.4). CORM was higher in accurate trials than in inaccurate ones (Fig. 8d) indicating that refixations are separated by more intervening fixations in accurate than inaccurate trials. CORM did not show an effect of target condition. There was no interaction between accuracy and target condition for any of the measures.

To ensure that the distance criterion of 2° was suitable for our dataset, we tested the dependence of our results on the criterion. The distance criterion determines the number of recurring fixations counted for each fixation location. Therefore, at a distance criterion of 0°, no fixation would be recurring with any other fixation, yielding a recurrence value of 0, whereas at a criterion of around 16° (half the size of the display), every fixation would recur with every other fixation leading to a recurrence value of 100%. For encircled stimuli similar to ours, Körner and Gilchrist, (2007) found less than chance discrimination of stimuli from 3° onward. Therefore, we varied the distance criterion from 0.5° to 3° in 0.5° increments and repeated the above analyses at each distance criterion for each recurrence measure. F-values for the effects of target and accuracy conditions at each radius increment are shown in Fig. S3. At 0.5°, we did not have recurrence values, therefore we only considered F-values at 1°, 1.5°, 2°, 2.5°, and 3°. There was an effect of target condition for all radii for recurrence (all *p* < 0.001) (Fig. S3A). In addition, an accuracy effect was seen at 1° (*p* = 0.048). There was an effect of target condition for both determinism (all *p* < 0.05) and laminarity (all *p* < 0.001) measures for radii 1.5°–3° (Fig. S3B, C). There is an effect of accuracy for the CORM measure from 1.5°–3° radius criterion (Fig. S3D). The near-constancy of our results around the distance criterion of 2° implies that our distance criterion has not biased the results.

## Discussion

In a task involving search and encoding of multiple targets among distractors for subsequent change detection, we explored changes in refixation patterns during memory encoding under various memory load conditions. Memory load was quantified by the number of targets in the search display. The subsequent change detection task tested accuracy for target memory. We considered refixations only during the 10-s *search* interval. For analysis of refixations, we applied two approaches. The first approach considered refixations relative to the item locations on the display. We called this approach *item-based refixation analysis*. We distinguished continued refixations, revisits and continued revisits, depending on whether the gaze left the boundary of a 2° circle around an item before refixating. The second approach involved *recurrence analysis* of refixation patterns. We analyzed refixations irrespective of proximity of fixation to an item by applying quantification measures to recurrence plots of fixations.

In the item-based refixation analysis, the number of targets presented in a trial affected target and distractor refixations in opposite manners. While targets showed greater number of refixations with increase in number of targets, i.e., with a larger memory load, distractors showed the opposite effect (Fig. 6). This reflects a shift in oculomotor strategy with increase in memory load as explained below. The increased refixations on targets with increase in memory load confirms the findings of earlier studies (Körner & Gilchrist, 2007; McCarley et al., 2003; Peterson et al., 2008). The opposite tendencies in the numbers of refixations on targets and on distractors have not been reported before. Target and distractor refixations have not been previously studied separately alongside controlled increase of memory load in a search task. Since the number of items per display is constant for the three target conditions, the increase in target refixations with simultaneous decrease in distractor refixations with memory load could be considered a trivial consequence of the change in the target-distractor ratio: as the number of targets increases in a display, the number of distractors decreases by a corresponding number. However, since we corrected the refixation counts based on the number of targets and distractors in the trial, the differences in refixations for targets and distractors are likely caused by a change in oculomotor strategy with increase of memory load.

Oculomotor strategy depends on the type of search task (Ballard & Hayhoe, 2009; Dickinson & Zelinsky, 2007). In previous studies on multiple target search, the task was either to indicate the presence of one or two targets in the search display (Körner & Gilchrist, 2007) or to determine if at least a certain number of targets was present in the display (Horowitz & Wolfe, 2001; McCarley et al., 2006; Takeda, 2002), requiring participants to identify targets, remember their locations, and count them. However, our multi-target visual search was more complicated since it was followed by a memory test and likely involved several sub-tasks: finding new targets, memorization of their orientations, keeping both location and orientation of targets in memory through the course of 10 s. All these task components may require refixations. Whereas target revisits are most likely made for rehearsal of their orientation, in accordance to the task demands, distractor revisits are likely caused by forgetting of already visited distractor locations while searching for targets. As mentioned in the *Introduction*, items may be insufficiently processed under enhanced working memory load (Gilchrist & Harvey, 2000; Gilchrist et al., 2001; Peterson et al., 2001). With an increase in memory load, more forgetting or insufficient processing of distractors and increased distractor revisits would be expected. But our results were opposite (Fig. 6d-f). Owing to limited central resources, participants likely faced a tradeoff between searching for new targets and rehearsing the orientation of targets already found during a trial. The observed opposite trend in target and distractor refixations may therefore reflect an oculomotor strategy employed to deal with said tradeoff. Since participants did not know the exact number of targets presented in a display, they would have to juggle between (1) continuing visual search for more targets, which involves revisiting forgotten or insufficiently processed distractors for identity discrimination and (2) revisiting targets for rehearsal. Specifically, in the three- and four-target conditions, after finding three or four targets, respectively, participants are likely to continue search for the possibly remaining targets. However, in the four- and five-target conditions, after finding four targets, there might be some “search fatigue,” resulting in an oculomotor strategy involving less search and more revisits to already known targets. Such search tendencies would result in more target refixations and less distractor refixations with increasing memory load, as is seen in our results (Fig. 6).

### Refixation types

We distinguished three types of refixations: continued refixations, revisits and continued revisits. Both continued refixations and continued revisits are successive fixations with short preceding saccades. Since a large amount of fixations facilitates memorization (Loftus, 1972), continued refixations and continued revisits are likely to be indicators of volitional scrutiny of an item, in particular, for encoding target orientation into visual working memory. For distractors, once identity has been established, only their location needs to be remembered. On this reasoning, while establishing identity may involve continued refixations, continued revisits are likely spurious for distractors. However, we must add here that continued refixations or revisits could also be a result of undershooting of saccades to the preferred viewing location, which is the center of words or objects (McConkie, Kerr, Reddix, & Zola, 1988; Nuthmann & Henderson, 2010). In this case, they could be corrective saccades to target locations. However, only the first continued refixation or revisit in a series could result from a corrective saccade. Therefore, undershooting of saccades is unlikely to be the complete explanation for continued refixations and continued revisits.

Revisits, on the other hand, have long preceding saccades not typically found in scrutinizing behavior. Revisits may therefore reflect attempts to compensate for loss of item information in working memory. Such losses may occur because of insufficient initial processing (Gilchrist & Harvey, 2000; Peterson et al., 2001) or interference from subsequently visited items (Zelinsky et al., 2011). In order to perform the task, both target location and orientation have to be remembered. As successive targets are memorized, they interfere with existing items in working memory prompting refixation of the decaying item – a monitor-refixate-rehearsal loop (Zelinsky et al., 2011). Because of the difference in size of the preceding saccades, revisits on targets are likely to have contrasting functions from continued revisits. They may be associated, in particular, with target location. Also, for distractors, revisits may, in principle, occur to compensate for forgetting their location.

We found no difference between continued refixations and continued revisits for targets (Fig. 6a, c). This result could, in principle, have two explanations. The first explanation is that both continued refixations and continued revisits are simply corrective saccades caused by saccadic undershoot. However, there were more continued refixations than continued revisits for distractors (Fig. 6d, f). This indicates that continued refixations and continued revisits are not mere corrective saccades. The second explanation is that later visits have equal importance for encoding as initial ones. This is in line with the result of our EEG eye-movement co-registration study of refixations, where we found that information acquisition manifests similarly for initial fixations and refixations (Nikolaev, Meghanathan, & van Leeuwen, 2018). The difference in continued refixations and continued revisits for distractors also confirms our suggestion that while distractors were scrutinized for encoding both location and identity in the first visit, in subsequent visits they were not further scrutinized. This evidence is corroborated by comparing target and distractor revisits to those in the random model (Fig. 7b, e). Target revisits were much more numerous than in the random model, implying a deliberate attempt to fixate on targets for rehearsal, whereas distractor revisits were very close to those in the random model. Note that there was an increase in distractor revisits with time both in the actual and model data, which could be attributed to the increased likelihood of fixation on a distractor as visual search progressed. Both target and distractor representations likely decayed once their identity had been established. While distractor revisits were triggered only during subsequent visual search, revisits to targets were triggered for rehearsal.

Task performance was affected by differences in target and distractor refixations. There were more revisits and continued revisits on targets in accurate than inaccurate condition (Fig. 6b). This indicates that performance improves when targets are revisited more and when there is greater scrutiny during rehearsal of targets. For distractors, meanwhile, the opposite occurred, that is, more revisits were observed in inaccurate than accurate condition (Fig. 6e). Also, there were more continued refixations on distractors in inaccurate than accurate condition. This result implies that when more time was spent encoding distractors or more revisits were made to distractors, performance deteriorated. These results delineate a clear strategy for better performance in the task – more rehearsal of targets via revisits and less time spent revisiting or scrutinizing distractors. However, there was no difference between accurate and inaccurate conditions for continued refixations on targets. This result is puzzling since more scrutiny and correspondingly better encoding would be expected for an improved performance.

A possible answer to this puzzle is provided by the results of the analysis of refixations over ten 1-s time windows during the visual search interval (Fig. 7). As seen from the decreasing trend of continued refixations with time, participants encoded targets and distractors more toward the beginning of the trial than toward the end. Both revisits and continued revisits increased for both targets and distractors, indicating that participants rehearsed targets and revisited forgotten distractor locations more over the course of a trial. This may imply that rehearsal of targets, and, therefore, maintenance of target representations in working memory was more important for performance in the task than initial encoding.

Our results suggest that performance may depend on recency of fixations in relation to the memory test. A memory benefit for recently fixated items during serial fixation of objects has been shown before (Irwin & Zelinsky, 2002; Zelinsky & Loschky, 2005). For example, Körner and Gilchrist (2007) asked participants to search the same display twice consecutively for two different targets. They found that fixation on the target in the second display was facilitated by fixation on the same letter in the first display for a recency of up to four fixations. This indicates a *recency effect* on memory for previously fixated targets in visual search. Similarly, recency of refixations rather than scrutiny of items may explain performance in our task.

The difference between targets and distractors that we have seen so far indicate that participants adapt their oculomotor strategies with increase in memory load. As memory load increases, performance improves if participants tailor their strategy to rehearsing more targets instead of revisiting forgotten distractors. This assertion is confirmed by the results of the recurrence analysis. The recurrence quantification measures provide insights into the nature of eye movement strategies employed by participants.

### Recurrence measures

Recurrence measures greater than chance show that eye movements in visual search definitely do not follow a random walk process. One prominent result is the increase of recurrence and laminarity with memory load, which concurs with the refixation results. This is not surprising since refixation count is a subset of the recurrence score. Meanwhile, laminarity, i.e., a measure of repeated fixation clusters, could be understood as volitional scrutiny akin to continued refixations and continued revisits. Therefore, an increase in laminarity with memory load suggests that the higher the working memory load, the more scrutiny of items during encoding. Note that, in recurrence analysis, we did not distinguish between targets and distractors. Therefore, the increase in recurrence and laminarity indicates an overall increase in refixations and scrutinizing behavior with an increase in memory load. This validates our recurrence analysis since the results are in line with those from the refixation analysis, in spite of the independence from target and distractor locations. The increase in recurrence and laminarity with memory load is in alignment with the “monitor-refixate-rehearse” mechanism (Zelinsky et al., 2011). As more items are held in working memory, there is increased decay of already-visited items and, therefore, increased revisits to these items.

Another prominent finding was the higher determinism in the five-target than the three- and four-target conditions indicating more repeated trajectories in the five-target condition. Thus, rehearsal of the scan-path is more prominent when working memory is being loaded to capacity (five items, reviewed in Luck & Vogel, 2013). Rehearsal of the scan-path is known to enhance recall of the memorized locations (Tremblay et al., 2006). Participants repeat, during retrieval, fixation sequences they performed during encoding (Foulsham, Walker, & Kingstone, 2009; Valuch et al., 2013; Wynn et al., 2016). Moreover, memory for scan-paths facilitates information retrieval (Bochynska & Laeng, 2015). Therefore, repetition of fixation sequences may be used as a mechanism for encoding targets in memory. This implies that a “monitor-refixate-rehearse” mechanism (Zelinsky et al., 2011) does not explain our results sufficiently. Increasing determinism with increasing memory load is evidence that participants encode not only the items, but also a path tracing item locations. Therefore, the nature of the working memory representations of visited items is complex. The traditional dichotomy of a memory representation being made up of identity and location of the item (reviewed in Baddeley & Logie, 1999; Beck et al., 2006; Smith & Jonides, 1997) is not sufficient to understand our results. It appears that in addition to identity and location, eye-movement sequences, or scan-paths, are also part of working-memory representations. This proposal resonates with earlier suggestions that oculomotor signals themselves maybe stored in working memory (Theeuwes, Belopolsky, & Olivers, 2009), and, more broadly, with ideomotor theory (Hommel et al., 2001).

On the other hand, it is known that serial order can be encoded in working memory (Ginsburg, Archambeau, van Dijck, Chetail, & Gevers, 2017; Hurlstone & Hitch, 2018; Marshuetz, 2005). Thus, it would follow that information about serial order of fixations is stored as a sequence of events and not as an oculomotor trace. It is impossible to behaviorally dissociate these two interpretations, since fixation order information will always overlap with eye-movement sequences.

Yet, the distinction between serial order information in fixation sequences and the oculomotor signal itself is an important one. Foulsham and Kingstone (2013) showed through a set of five experiments involving encoding and recognition tasks for natural scenes that re-presentation of previously seen locations enhances memory for those locations, whereas re-presentation of fixation order does not facilitate recognition memory. In their experiments, however, memory for fixation order was tested by serial presentation of patches (parts) of visual stimuli either in the presentation stage or in the recognition stage. This would seriously disrupt any oculomotor memory trace, and may explain why they did not find that serial order information facilitated recognition. Taking into account the overlap between the oculomotor and serial order components of fixation sequences, it is reasonable to propose that an oculomotor component exists in VWM as we explain below.

Eye movements are closely tied to visuo-spatial working memory. Both eye movements and shifts in spatial attention interfere with maintenance of information in working memory (Lawrence, Myerson, & Abrams, 2004; Smyth, 1996; Smyth & Pelky, 1992) and spatial memory span (Pearson & Sahraie, 2003). Maintenance of spatial information is accomplished by shifts in spatial attention (Awh & Jonides, 2001; Smyth & Scholey, 1996). We also know that attention is shifted covertly to a location before an eye movement is made to that location (Deubel & Schneider, 1996; Hoffman & Subramaniam, 1995; Kowler, Anderson, Dosher, & Blaser, 1995). Therefore, spatial attention rather than eye movement per se may be affecting working memory. However, an effect of eye movements on spatial information is seen as distinct from that of spatial attention (Lawrence et al., 2004; Theeuwes et al., 2009; Tremblay et al., 2006). When participants were to memorize the locations of two items only through shifting of covert attention, their performance was lower than when eye movements were made (Lawrence et al., 2004). Since eye movements contribute to better performance in a test of spatial memory, it is likely that they are also a part of visuo-spatial representations in working memory, which is what our results imply.

Eye movements need not be the only motor actions that affect working memory. Limb movements affect maintenance of spatial information (Pearson & Sahraie, 2003; Smyth, Pearson, & Pendleton, 1988). This may imply that working memory incorporates motor information. Working memory functions emerge via attention by coordination of sensory and action related functions (Postle, 2006; Theeuwes et al., 2009). For instance, it is a crucial principle of *grounded cognition* (Barsalou, 2008) that retrieval takes place through *simulation*, i.e., the reenactment of perceptual, sensory, and motor states acquired during experience.

If eye movements are a part of working memory representation and can be reenacted, participants would be capable of incorporating a number of strategies for maintenance of information in working memory. Our results provide evidence for multiple strategies participants employ to perform our task. As memory load increases, participants make (1) fewer distractor refixations and more target refixations, (2) more fixation clusters, and (3) more repeated fixation sequences.

We can view all of the above strategies as rehearsal via reenactment of earlier fixations. As memory load increases, participants reenact target fixations more frequently than distractor fixations. Repeated fixation clusters are reenactment of encoding the items. Fixation sequences, when repeated, are simulations of entire working memory representations of items, which is rehearsal of these items. We observed a difference between original fixations and refixations in EEG amplitudes during the presaccadic interval in our EEG eye-movement co-registration study (Nikolaev et al., 2018). Since the presaccadic interval may reflect oculomotor planning (Csibra, Johnson, & Tucker, 1997; Richards, 2003), it is likely that this difference between fixations and refixations highlights the difference between encoding into working memory and reenactment of this process. At the same time, it is likely that this type of refixation behavior itself is triggered by monitoring activation levels of working memory representations. We could conceive of a “monitor-reenact-rehearse” mechanism for maintaining information in working memory instead of a “monitor-refixate-rehearse” mechanism (Zelinsky et al., 2011) to explain the richness of the strategies participants used in our task.

An advantage of storing eye movements in working memory is that they may convey information about the relationship between items, for example, relative position, orientation, or identity. Rehearsal of fixation sequences may bring such relational information to expression in working memory. Relational information is also present in the frequency of refixations, which is described by our final recurrence measure, CORM. CORM quantifies the temporal interval between the first fixation and refixation on a particular location. There was no effect of memory load on CORM. In turn, CORM showed quicker refixations (i.e., shorter intervals between refixation sequences) in the inaccurate than accurate condition. This implies that participants performed better when they refixated items after longer intervals. This result complements that found about more refixations on distractors in the inaccurate than accurate condition. This means that when there were quicker revisits to previously fixated items and more distractor scrutiny and revisits, performance was worse. Quicker revisits are likely to be a symptom of insufficient attention during the task. Premature attention shifts may promote quick revisits, in particular with increased memory load (Gilchrist & Harvey, 2000; Gilchrist et al., 2001; Peterson et al., 2001), leading to inefficient processing of previously fixated items (Dickinson & Zelinsky, 2007; Henderson, 1992; Peterson et al., 2001). On the other hand, when items are revisited before their working-memory representations have sufficiently decayed, this adds no benefit to these items, while it leaves less time for visiting new ones. For either of these reasons, an oculomotor strategy involving quick refixations of items is associated with poorer performance.

Even though we have pitted the item-based refixation analysis and recurrence analysis against each other as complementary methods in this paper, the opposition is only constructed. In principle, recurrence plots could be used to analyze refixations specifically on targets or distractors (Anderson et al., 2013) and could also be adapted to different refixation types. The advantage of using recurrence plots without attribution to item locations is that the number of assumptions made about fixations is relatively less compared to the item-based refixation analysis. Specifically, to compute recurrence measures, we had to choose only two criteria: a distance of 2° around a fixation and a minimal length of two for lines on the recurrence plot (see Methods). Whereas, for the refixation analysis, classification of a fixation into one of the three refixation types on a target or a distractor may involve ambiguous situations, which may have led to rare misclassifications. For instance, consider the sequence of three fixations around a target: (1) fixation 1.5° from target, (2) fixation 2.2° from target, and (3) fixation 1.7° from target. Here, fixations 2 and 3 would not get classified as target fixations or refixations at all, when, in reality, it is highly likely that they both are continued refixations on the target. In this respect, the recurrence analysis provides reprieve in that it is much more robust to distance criteria as we saw in the results. Additionally, recurrence analysis offers the possibility of studying and comparing scan-paths, which may be as important for eye-movement behavior as identity and location of items. Together, the item-based refixation analysis and recurrence analysis have given us insights into different aspects of refixation behavior. While the item-based refixation analysis distinguished differences between encoding of targets and distractors, recurrence analysis identified rehearsal of items in memory through refixation patterns.

In our study, though we find scan-path repetitions, the evidence for scan-paths being stored in VWM is only corroborative. The interpretation of our results has relied heavily on previous studies showing scan-path repetition for rehearsal of items (Brandt & Stark, 1997; Johansson & Johansson, 2014) and modeling of a VWM component to explain refixation behavior (Zelinsky et al., 2011). To show that scan-paths are indeed part of VWM, an experiment needs to (1) dissociate the order information component from the oculomotor component in eye-fixation sequences and (2) ensure that scan-path repetition cannot be explained exclusively by other refixation rules like distance, IOR, and so on. Future studies and modeling are required to rule out alternative explanations for scan-path repetitions. As a first step, in this paper, we present the idea that scan-path repetitions during encoding of items are part of active rehearsal to store oculomotor traces in VWM.

## Conclusion

In our multi-target visual search task, performance was better on three occasions: (1) if participants revisited more targets instead of distractors, (2) if participants revisited items after long intervals, and (3) if participants visited targets toward the end of the trial. Overall, the implications are that performance in our task was better when the refixation strategy involved more target revisits, widely spaced fixation sequences (or better attention) and revisits toward the end of search. We were able to identify these strategies by studying fixation patterns, which revealed that scan-paths or fixation sequences were a part of working memory representation. The modal nature of representations in working memory, which involves oculomotor function, affords the use of a number of strategies for information encoding and maintenance. Participants could strategically reenact subsets of the encoding process in order to maintain acquired item information. Participants could choose to revisit more targets instead of distractors, they could repeat sequences of fixations and could choose when to do them in order to successfully maintain information. We conclude that scan-paths are a part of working-memory representations.

## Electronic supplementary material

ESM 1 (DOCX 289 kb)

ESM 2 (DOCX 12 kb)

## Footnotes

1 Mean, uncorrected target fixation counts for three-, four-, and five-target conditions were 8.9, 12.1, and 15.0 respectively. Uncorrected target fixation numbers increased (F(2, 38) = 363.6, p < 0.001, η^2^ = 0.95) with number of presented targets (all *post hoc* p < 0.001), whereas distractor fixations decreased (F(2, 38) = 596.4, p < 0.001, η2 = 0.97) with the number of presented targets (all *post hoc* p < 0.001) with means of 30.5, 26.4, and 22.5 in three-, four-, and five-target conditions, respectively.
