# Supplementary material for: Refixation patterns reveal memory-encoding strategies in free viewing
Source: Atten Percept Psychophys. 2019 May 1;81(7):2499–516. doi: 10.3758/s13414-019-01735-2 (PMC6848043; doi:10.3758/s13414-019-01735-2)
Supplement: Supplementary file 1 — (DOCX 289 kb) [file 13414_2019_1735_MOESM1_ESM.docx]

Supplementary Material


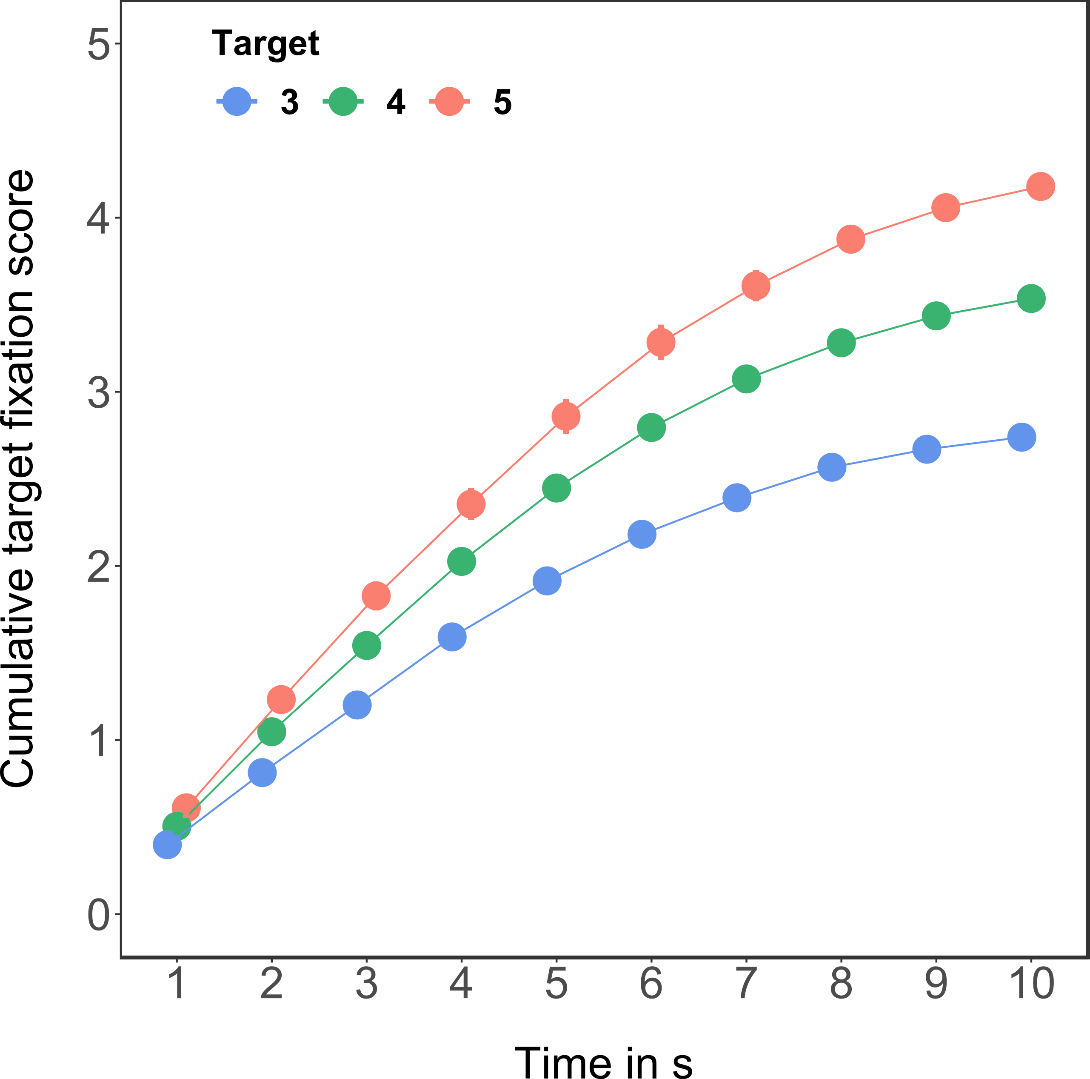


*Figure S1*. Average cumulative target fixation scores for 20 participants in 1-s time intervals during 10 s search display for 3-, 4- and 5-target conditions.


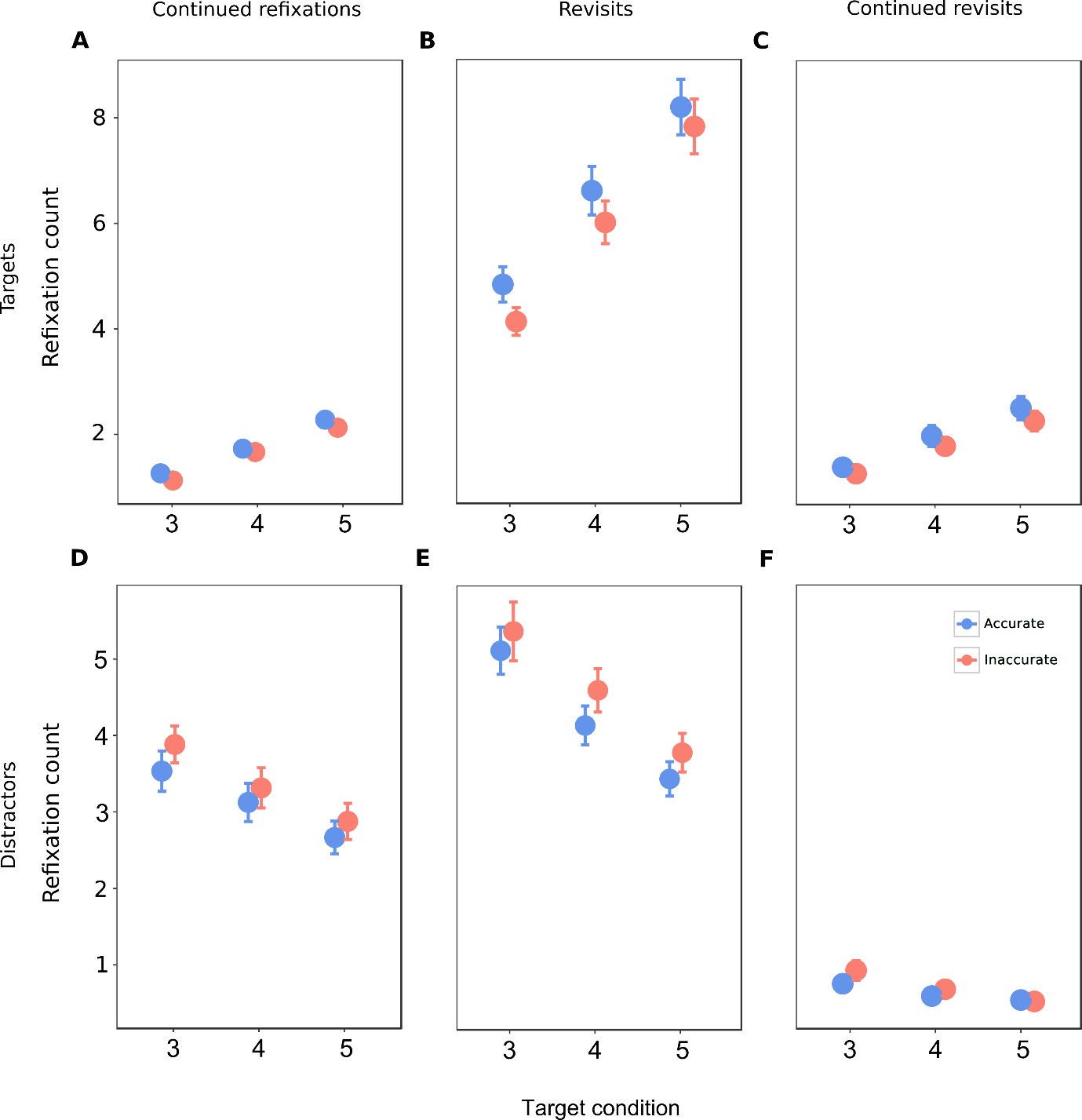


*Figure S2.* Three types of refixations in 3-, 4- and 5-target conditions. Uncorrected count of A: continued refixations on targets, B: revisits on targets, C: continued revisits on targets, D: continued refixations on distractors, E: revisits on distractors and F: continued revisits on distractors. The 3-, 4- and 5-target conditions are along the x-axis. Data points are the means and error bars are the standard errors across 20 participants.


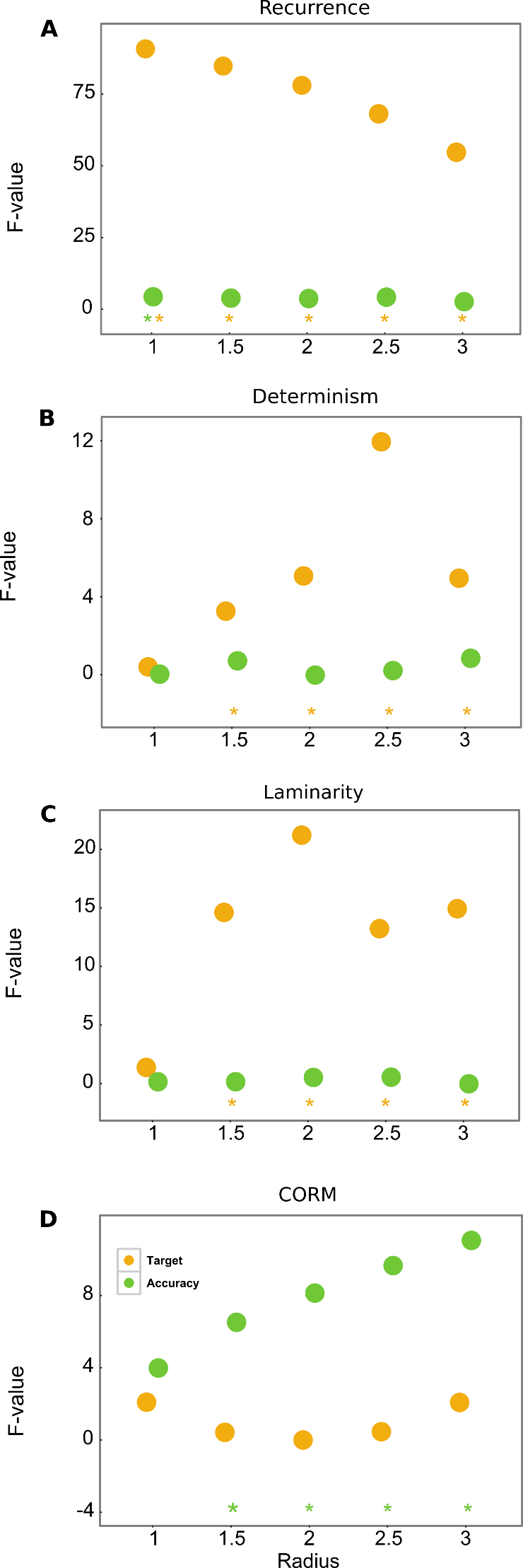


*Figure S3.* Effect of distance criterion. F-values for effect of number of targets and accuracy on A: Recurrence, B: Determinism, C: Laminarity and D: CORM, for radii 1°, 1.5°, 2°, 2.5° and 3° of visual angle, as distance criteria. The asterisks indicate significant effects for target (yellow) and accuracy (green).
