# Supplementary material for: Refixation patterns reveal memory-encoding strategies in free viewing
Source: Atten Percept Psychophys. 2019 May 1;81(7):2499–516. doi: 10.3758/s13414-019-01735-2 (PMC6848043; doi:10.3758/s13414-019-01735-2)
Supplement: Supplementary file 2 — (DOCX 12 kb) [file 13414_2019_1735_MOESM2_ESM.docx]

1 - Mean, uncorrected target fixation counts for 3-, 4- and 5- target conditions were 8.9, 12.1 and 15.0 respectively. Uncorrected target fixation numbers increased (F(2, 38) = 363.6, p < 0.001, η^2^ = 0.95) with number of presented targets (all post-hoc p < 0.001). Whereas, distractor fixations decreased (F(2, 38) = 596.4, p < 0.001, η2 = 0.97) with the number of presented targets (all post-hoc p < 0.001) with means of 30.5, 26.4 and 22.5 in 3-, 4- and 5-target conditions, respectively.
